# A Comparison Between Cross-Linking Protocols in Patients With Progressive Keratoconus: A Review

**DOI:** 10.7759/cureus.31029

**Published:** 2022-11-02

**Authors:** Ahmed A Aldayel, Haifa M Alwael, Reem M Alshathri, Hebah A Alnasser, Lama A Alfawzan

**Affiliations:** 1 Ophthalmology, King Khaled Eye Specialist Hospital, Riyadh, SAU; 2 Medicine and Surgery, King Saud University, Riyadh, SAU

**Keywords:** conventional collagen crosslinking, accelerated collagen crosslinking, epithelial-off, trans-epithelial, keratoconus, corneal collagen cross linking (cxl)

## Abstract

Keratoconus (KC) is a noninflammatory cornea disease leading to progressive thinning, causing it to change from its normal dome shape to a cone shape. One of the novel treatments of KC is corneal collagen cross-linking (CXL). Due to its importance, many studies have been conducted to compare corneal cross-linking protocols; this review article aims to discuss corneal cross-linking and compare its different treatment options, including Dresden, accelerated, and customized protocols in patients with progressive KC and their respective long-term outcomes. A search was performed in PubMed and Google Scholar with no language, dates, or study type restriction. Most of the results showed almost no difference between protocols over traditional cross-linking. However, published data are limited, long-term outcomes of novel age groups remain unclear, and further studies are needed.

## Introduction and background

Keratoconus (KC) is a noninflammatory, mostly bilateral, degenerative disorder of the cornea characterized by progressive ectasia and stromal thinning [1]. KC is the most common cause of corneal transportation [1], and it usually begins in late childhood to early adulthood and progresses in severity until age 35 to 40 years [2]. 

The worldwide prevalence of reported cases of KC varies widely depending on multiple factors such as geographic location, diagnostic criteria used, and the type of patients selected [3]. For example, KC in the USA occurs in 500 in 100,000 patients, while in India, the prevalence is 2,300 in 100,000, and in China, the prevalence is 27 in 100,000 patients [2]. In 2009, a single-center study at King Khaled Eye Specialist Hospital in Riyadh, Saudi Arabia, demonstrated KC's prevalence in different provinces. KC had the highest prevalence in the Central region (38.09%), whereas the Southern, Northern, Western, and Eastern had 37.5%, 10.14%, 8.86%, and 5.41%, respectively [4]. Another study was done on 522 patients in Riyadh, Saudi Arabia, which showed a prevalence of 4.79% with a ratio of 1:21 patients [5]. However, more studies are needed on the prevalence of KC in Saudi Arabia.

This review article focuses mainly on corneal collagen cross-linking (CXL) and its different protocols in patients with progressive KC and their respective long-term outcomes. A search was performed in PubMed and Google Scholar with no language, dates, or study type restrictions. The keywords progressive KC, conventional cross-linking, accelerated cross-linking, customized cross-linking, topography-guided cross-linking, and trans-epithelial vs. epithelium-off CXL were used, and titles and abstracts were screened for relevance. 

## Review

Definition

CXL is an invasive method for altering the stromal structure of the cornea used for the management of KC. This method depends on the synergy between ultraviolet A (UVA) light (370 nm) and topical riboflavin (vitamin B) [1]. A 1997 CXL study compared riboflavin with UV light or glutaraldehyde or Karnovsky's solution in the treatment of KC against other treatment options. Results showed that CXL treatment led to increased corneal stiffness, and follow-up showed that the progression of KC stopped in all patients along with regression in keratometric and refractive values [6]. The CXL works mainly by the formation of chemical bonds between collagen fibrils. Therefore, it strengthens the cornea and prevents disease progression [1].

Diagnosis

KC often goes undiagnosed early in the disease until significant progression has occurred [3]. Therefore, KC should be suspected in patients with significant irregular astigmatism that is unstable and rapidly increasing. In advanced cases, the cone-shaped protrusion can be visible on a slit lamp as progressive corneal thinning and distortion have occurred [7].

Multiple diagnostic modalities to aid in the diagnosis of KC have been developed over the last decades, including corneal topography and tomography, KC indices, the Belin Ambrosio Enhanced Ectasia Display, Holladay six-map display, corneal pachymetry, automated screening program, and corneal biomechanics [7].

Management

The choice of management options for KC is based on its severity and progression and the visual demands of the patient [8]. Management options are centered on two main domains: prevention of disease progression and improvement of vision. Under each domain, management options can be broadly divided into surgical and nonsurgical interventions. Nonsurgical options that aim to improve vision include spectacles, soft contact lenses (CLs), and rigid gas permeable CLs. In patients who cannot tolerate a CL due to poor CL fitting or poor vision due to corneal scar, surgical options for vision correction are considered with corneal rings or keratoplasty [9]. Spectacles and various forms of CLs are the preferred line of management in the early course of the disease, as corneal astigmatism and asymmetry between the two eyes are not significant [8].

Prevention of risk factors is essential during treatment, including avoiding allergens and eye rubbing [10]. In terms of halting the progression of the disease, one novel technique is CXL [8]. This technique has been developed based on understanding the pathophysiology of KC progression.

Outcomes of cross-linking protocols

Accelerated vs. Conventional CXL

The traditional cross-linking protocol is also known as "the Dresden protocol" and "standard protocol," which consist of several techniques as follows: Use of riboflavin 5-phosphate and dextran solution to the de-epithelized surface every five minutes for 30 minutes, then application of UVA 370 nm, 3 mW/cm2 for 30 minutes for the total energy of 5.4 J/cm2 with the application of riboflavin and dextran every five minutes [6]. However, due to the long duration of the procedure, current research focuses on the accelerated cross-linking protocol as a possible way to shorten the duration of the procedure using the concept of an interchangeable amount of power with the same total energy of 7.2 J/cm2 [11,12]. This can be achieved by increasing the illumination intensity, also known as accelerated cross-linking.

Comparative studies in the literature report varying results when comparing the two protocols, but most studies agree that accelerated and conventional cross-linking yield comparable or equivalent results. This review highlights the significant differences found in the literature among these two treatment modalities and further divulges notable and statistically significant results.

Cınar et al. compared the two treatment modalities in parameters such as visual and refractive keratometric outcomes in patients with progressive KC [1]. They defined progressive KC as loss of one or more lines in best-corrected distance visual acuity, or one or more diopters or more increment in maximum keratometry (Kmax). Their six-month study included 26 patients. Change in uncorrected visual acuity (UCVA) and corrected distance visual acuity showed a statistically significant improvement in the accelerated group (p = 0.035 and p = 0.047, respectively) compared to the conventional group. However, the results did not yield any statistically significant improvements in the other parameters [1].

The trend was further supported by a comparative study by Males et al. in Australia on 42 eyes [2]. They concluded that accelerated and conventional cross-linking were equally effective in stabilizing progressive KC with no statistically significant differences in corneal curvature, visual acuity, and corneal thickness in their 12-month study [2].

Further findings of accelerated CXL include a favorable outcome in halting KC progression. A six-month study conducted in Turkey on 23 patients showed a significant improvement in best-corrected distant visual acuity [3]. The authors reported that best-corrected distant visual acuity improved from 0.49 logMAR to 0.34 logMAR (p=0.026), and they reported improvements in the mean cylinder (p=0.002), spherical equivalent (p=0.045), flat keratometry, steep keratometry, mean keratometry, and Kmax (p = 0.025, p < 0.001, p = 0.004, and p = 0.03, respectively) [3].

Long-term results of accelerated CXL at 6 mW/cm2 with 365-nm wavelength (UVA) for 15 minutes in patients with progressive KC, defined by an increase by 1D of astigmatism, myopia, or Kmax, were assessed in a study conducted by Waszczykowska et al. in Poland on 16 patients [13]. In 18.7% of patients with a higher preoperative Kmax value (>50 D), the authors found significant cornea flattening. Alternatively, steepening of the cornea occurred in one patient (6.25%) with a lower Kmax value (<50 D) in the 24-months study [13]. Wittig-Silva et al. conducted a 36-month study in Australia on 100 eyes to compare conventional CXL between control and treated groups [14]. This improved Kmax, UCVA, and best spectacle-corrected visual acuity in the treated group, while the patients in the control group experienced disease progression [14].

Few studies have demonstrated better outcomes via the standard treatment of progressive KC than accelerated CXL. For example, an 18-month randomized clinical trial conducted by Hashemi et al. in Iran on 31 eyes yielded better results in corneal flattering via standard therapy than accelerated CXL using 18 mW/m2 for five minutes, with statistically significant changes in keratometry parameters in the standard group [15]. However, various studies indicated the need for additional clinical trials of accelerated CXL with a longer follow-up to attest to such findings further.

Transepithelial vs. Epithelium-Off CXL

CXL surgical techniques can be categorized into an epithelium-off method (standard) and a transepithelial method (TE-CXL). The epithelium-off method relies on the debridement of corneal epithelium to enhance the penetration of riboflavin [16]. TE-CXL avoids corneal epithelium debridement by using alternative methods proposed in the literature to increase the penetration, including the use of chemical enhancers to riboflavin, mechanical disruption of the epithelium, increasing the duration of exposure to riboflavin, or the use of ultrasound [17].

Although CXL has proven effective in preventing disease progression, removing the epithelium remains controversial due to its adverse effects. Patients with thin corneas (as noted in some Asian patients) would be well suited for transepithelial CXL because thinner corneas lack the UVA protective qualities of intact endothelium, making them amenable to damage by UVA [18]. Given this, many studies have compared outcomes of the epithelium-off method with TE-CXL [19].

A 2021 prospective study of 64 eyes with progressive KC in Spain with a three-year follow-up compared epithelium-off and TE-CXL methods. They evaluated coma-like aberration, higher-order aberrations (HOAs), densitometry, Kmax, asphericity at 8 mm and 10 mm (Q8, Q10), pachymetry at the apex, pachymetry at the thinnest point, index of surface variance (ISV), and the minimum radius of curvature. After epithelium-off CXL, patients improved the coma-like aberration, HOAs, Q8, Q10, and ISV values, while after TE-CXL, these variables remained unchanged. Moreover, densitometry and corneal pachymetry improved significantly after epi-off CXL compared to TE-CXL. Kmax and the anterior minimum radius remained were unaffected by either treatment [19].

A 2016 randomized controlled trial in Russia involved 149 eyes with progressive KC with a two-year follow-up. The authors reported keratometry regression in both groups, but epithelium-off CXL was more effective than TE-CXL [20]. Another prospective clinical study in Italy published in 2016 involving 40 eyes with progressive KC with a one-year follow-up reported a significant reduction of Kmax in the epithelium-off CXL group, whereas the TE-CXL group curvature was not significantly affected [21]. Minimum pachymetry values were stable with the TE-CXL group, whereas the epithelium-off CXL group recorded a significant corneal thinning. However, both groups saw statistically significant aberrometric improvements [21]. A 2015 Saudi Arabian study contained 70 patients with progressive KC to demonstrate the efficacy of CXL in the presence or absence of corneal epithelium [22]. Thirty-four of these patients underwent TE-CXL, and 36 underwent CXL after removing the epithelium. The authors found that all patients who underwent epithelium-off CXL had better surgical outcomes than the TE-CXL group. In their study, 55% of the TE-CXL group progressed, and the rest of the patients experienced an increase in Kmax during the three-year follow-up period. The epithelium-off group had a significant decrease in Kmax [22]. A Turkish retrospective cohort study published in 2015 with an 18-month follow-up found that while TE-CXL seemed to have reduced effectiveness in inducing improvement in topographic indices, its effect on visual acuity is likely to be similar to that of epithelium-off CXL [23].

Customized Cross-Linking

Recent studies proposed that the biomechanical modification in KC is focal rather than a uniform generalized weakening of the cornea, creating a cycle of increased strain, stress redistribution, and subsequent focal steepening and thinning. This proposed theory serves as the base of the novel technique of customized corneal cross-linking (X-CXL), which selectively uses more energy in weaker areas of the cornea and leaves the stronger areas with little to no energy used [7]. X-CXL is a general term that includes multiple novel techniques, including accelerated topography-guided CXL, accelerated pachymetry-guided CXL, and accelerated CXL-Plus (a combination of CXL and refractive surgical techniques) [7]. For this review, we will focus on accelerated topography-guided CXL.

Seiler et al. conducted a one-year prospective study at the Institut für Refraktive und Ophthalmo-Chirurgie, in Zurich, Switzerland, that compared outcomes of standard (n=20) and X-CXL (n=20) treatments on 40 eyes of 40 patients with documented progressive primary KC [24]. They demonstrated that X-CXL had a shorter epithelial healing time, more significant change in Kmax (p=0.039), better regularization index of the cornea (p=0.03), and a more substantial flattening effect than standard CXL. However, the authors note that more extensive studies and longer follow-up times are needed before considering X-CXL as a routine clinical procedure [24].

At Kitasato University, a study was done with similar results [25]. It included 42 eyes undergoing epithelium-on, accelerated, oxygen-supplemented X-CXL to evaluate the combination of promising novel treatment strategies for progressive KC patients. Baseline and outcome follow-up measurements of refraction, visual acuity, and corneal tomography were taken at one, three, and six months and one year postoperatively. The authors found no noticeable progression of KC, with reduced Kmax (p<0.001 at one year), reduced astigmatism, and improved best spectacle-corrected visual acuity at one year (p=0.004), with an amenable safety and patient comfort profile and no significant adverse events [25].

The summary of all referenced articles is included in Table 1, in the Appendices.

## Conclusions

Many studies have compared CXL protocols with somewhat similar results. All such studies reported that CXL led to halting disease progression safely. The differences in the study outcomes seem to be geographically dependent. However, published data are limited in comparing different patient populations and age groups. Long-term outcomes of novel age groups remain unclear, and further studies are needed.

## Appendices

**Table 1:** Summarization of all referenced articles

Reference NO.	Study	Author	Year of Publication	Country	Comparison	Number of Participants	Follow up duration	Mean change in Uncorrected Visual Acuity (logMAR)	Mean change in Corrected visual Acuity (logMAR)	Mean change in Maximum keratometry (D)	Mean change in Mean keratometry (D)
1	Comparison of accelerated and conventional corneal collagen cross-linking for progressive keratoconus	Cınar Y	2013	Turkey	Accelerated vs. Conventional	26	6 months	Accelerated: -0.25 Conventional: -0.15 , p(0.408)	Accelerated: -0.19 Conventional: -0.08 , p(0.624)	Accelerated: -1.14D Conventional: -0.94D, p(0.739)	Accelerated: -0.65D Conventional: -0.45D , p(0.762)
2	Comparative study of long-term outcomes of accelerated and conventional collagen crosslinking for progressive keratoconus	Males JJ	2017	Australia	Accelerated vs. Conventional	42 eyes	12 months	Nill	Accelerated: −0.06 Conventional: −0.03, p(0.06)	Nill	K2:Accelerated: -1.11D Conventional: -0.61D , p(0.36)
3	Accelerated corneal collagen cross-linking for progressive keratoconus	Cınar Y	2013	Turkey	Accelerated	23	6 months	-0.21	-0.15 , p(0.026)	-1.35D , p(0.03)	-0.72D , p(0.04)
13	Two-Year Accelerated Corneal Cross-Linking Outcome in Patients with Progressive Keratoconus	Waszczykowska A	2015	Poland	Accelerated	16	24 months	no change	no change	-0.65D , p(>0.05)	Nill
14	A Randomized, Controlled Trial of Corneal Collagen Cross-Linking in Progressive Keratoconus	Wittig-Silva	2014	Australia	Conventional	100 eyes	36 months	-0.15 , p(0.001)	-0.09 , p(0.347)	-1.03D , p(<0.001)	Nill
15	Long-term Results of an Accelerated Corneal Cross-linking Protocol (18 mW/cm2) for the Treatment of Progressive Keratoconus	Hashemi H	2015	Iran	Accelerated vs. Conventional	31 eyes	18 months	Accelerated: -0.09 p(0.176) Conventional: -0.06 p(0.107)	Accelerated:no change p(0.451) Conventional: -0.01 , p(0..943 )	Accelerated: -0.06D p(0.407) Conventional: -0.64D, p(0.005)	Accelerated: -0.21D p(0.309)Conventional: -0.71D , p(0.004)
19	Epithelium-Off vs. transepithelial corneal collagen crosslinking in progressive keratoconus: 3 years of follow-up	Arance-Gil Á	2021	Spain	Epi-off vs. Trans-epi	64 eyes	36 months	Nill	Central KC: -0.2 Paracentral: -0.08 , p(0.001)	Epi-off: -0.81D Trans-epi: +0.6D , p(0.038)	Nill
22	Transepithelial Versus Epithelium-Off Corneal Collagen Cross-Linking for Progressive Keratoconus: A Prospective Randomized Controlled Trial	Mashhoor F Al Fayez	2015	Saudi Arabia	Epi-off vs. Trans-epi	70	3 years	Epi-off: -0.2 Trans-epi: +0.1 p(<0.0001)	Epi-off: -0.1 Trans-epi: +0.06 p(0.055)	Epi-off: more than 2.25D flattening Trans-epi: more than 0.75 steepening ,p(<0.0001)	Epi-off: 100% stability or improvement Trans-epi: 45% stabilized and 55% progressed ,p(<0.0001)
23	Transepithelial versus epithelium-off crosslinking in adults with progressive keratoconus	Çerman E	2015	Turkey	Epi-off vs. Trans-epi	60 eyes	18 months	Epi-off: - 0.12 p(< 0.001) Trans-epi: -0.12 p(< 0.001)	Epi-off: - 0.12 p(< 0.001) Trans-epi: -0.11 p(<0.001)	Epi-off: - 2.3D p(< 0.001) Trans-epi: -0.12 p(>0.05), p(<0.001)	Nill
24	Customized Corneal Cross-linking: One-Year Results	Seiler TG	2016	Switzerland	Customized CXL vs standared CXL	40	1 year	Nill	customized: -0.07 p(0.08) Standard: -0.04 p(0.31) , p(0.22)	Customized: -1.7D p(0.001) Standard: - 0.9D p(0.004), p(0.039)	Nill
21	Transepithelial Iontophoresis Versus Standard Corneal Collagen Cross-linking: 1-Year Results of a Prospective Clinical Study	Vinciguerra P	2016	italy	Transepithelial vs standard (S-CXL and I-CXL)	40	1 year	nill	trans-epi with Iontophoresis:-0.13 p(0.0003), Standard CXL: -0.8 p(0.03)	trans-epi with Iontophoresis:-0.86D p(0.44), Standard CXL: -1.05D p(0.005)	Nill
25	Visual and Topographic Improvement with Epithelium-On, Oxygen-Supplemented, Customized Corneal Cross-Linking for Progressive Keratoconus	Kamiya K	2020	Japan	Accelerated, Oxygen-Supplemented, Customized	42 eyes	1 year	pre-op:0.87 ± 0.53 ,post op :0.78 ± 0.56 p(0.016)	pre-op: 0.19 ± 0.36 , post op : 0.11 ± 0.33 ,p(0.004)	pre op:53.04 ± 7.91 D,post op :52.31 ± 7.50 D ,p(<0.001)	Nill
20	Standard corneal collagen crosslinking versus transepithelial iontophoresis-assisted corneal crosslinking, 24 months follow-up: randomized control trial	Bikbova G	2016	Russia	Standard vsTransepithelial	149 eyes	2 years	standard : -0.13 , Transepithelial CXL via iontophoresis :- -0.29	standard: - 0.02,Transepithelial CXL via iontophoresis :-0.07	Nill	standard: - 2.15D,Transepithelial CXL via iontophoresis :-0.97D
